# Are older people worse off in 2040 regarding health and resources to deal with it? - Future developments in complex health problems and in the availability of resources to manage health problems in the Netherlands

**DOI:** 10.3389/fpubh.2023.942526

**Published:** 2023-06-16

**Authors:** Fatiha Baâdoudi, Susan H. S. J. Picavet, Henk B. M. Hildrink, Roy Hendrikx, Mieke Rijken, Simone R. de Bruin

**Affiliations:** ^1^National Institute for Health and the Environment (RIVM), Bilthoven, Netherlands; ^2^Netherlands Institute for Health Services Research (Nivel), Utrecht, Netherlands; ^3^Department of Health and Wellbeing, Windesheim University of Applied Sciences, Zwolle, Netherlands

**Keywords:** health problems, older people, resources, health care, social care, health policy

## Abstract

**Introduction:**

Developing sustainable health policy requires an understanding of the future demand for health and social care. We explored the characteristics of the 65+ population in the Netherlands in 2020 and 2040, focusing on two factors that determine care needs: (1) the occurrence of complex health problems and (2) the availability of resources to manage health and care (e.g., health literacy, social support).

**Methods:**

Estimations of the occurrence of complex health problems and the availability of resources for 2020 were based on registry data and patient-reported data. Estimations for 2040 were based on (a) expected demographic developments, and (b) expert opinions using a two-stage Delphi study with 26 experts from policy making, practice and research in the field of health and social care.

**Results:**

The proportion of people aged 65+ with complex health problems and limited resources is expected to increase from 10% in 2020 to 12% in 2040 based on demographic developments, and to 22% in 2040 based on expert opinions. There was high consensus (>80%) that the proportion with complex health problems would be greater in 2040, and lower consensus (50%) on an increase of the proportion of those with limited resources. Developments that are expected to drive the future changes refer to changes in multimorbidity and in psychosocial status (e.g., more loneliness).

**Conclusion:**

The expected increased proportion of people aged 65+ with complex health problems and limited resources together with the expected health and social care workforce shortages represent large challenges for public health and social care policy.

## Introduction

Demographic projections show that in most Western countries the proportion of the population aged 65+ will increase in the coming decades (1–3). In the Netherlands for instance, the proportion of people of 65 years or older is expected to increase from 20% in 2020 to 26% in 2040 (4, 5). Within this age group, the percentage of people over age 80 and 90 will increase too. Older people generally have increased health and social care needs, and thus these demographic changes will have wide-ranging implications for society as a whole and for health and social care provision in particular (6). In addition to these demographic developments, also other developments may affect how the 65+ population of 2040 will look like with regard their demand for health and care, compared to those in 2020. Insight into these developments is needed for governments to design sustainable health policy and explore policy options.

It is expected that in the future people over age 65 will differ from their predecessors, e.g., in terms of lifestyle, health literacy, digital skills, household composition and social networks. For example, it is thought that the prevalence of smoking will continue to decrease, resulting in less smoking-related health problems. Overweight and obesity, however, still seem to increase, which implies higher numbers of overweight-related health problems (5, 7–9). Besides these health-related developments, we live in an era in which profound cultural, social and economic changes are taken place that will determining the future (10, 11). Since this “package deal of changes” includes many different elements it is difficult to determine the consequences for future health and social care needs (6, 12).

**Figure 1:**
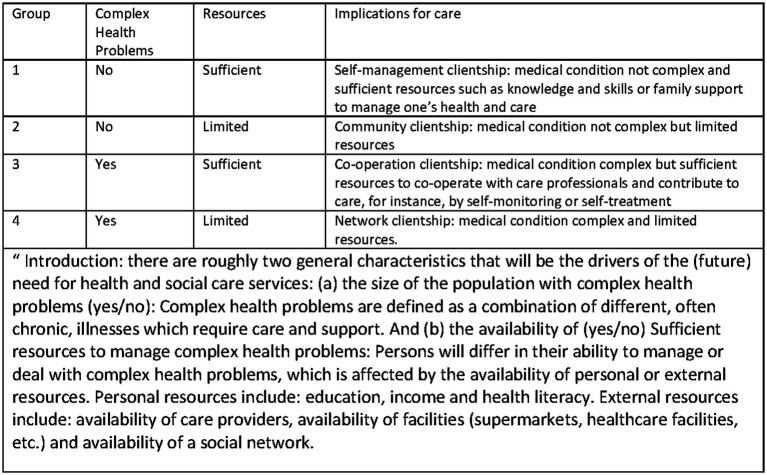
The ‘care and support’ groups in the population of the thinking model used, and the introduction to the experts. Based on the clientship model (13, 15, 16).

One way to get more insight into the consequences of a changing 65+ population is to focus on two important elements that determine the need for care and support: (1) the complexity of the medical condition(s), − which we refer to as ‘complex health problems’, and (2) the availability of resources for managing health and care (13, 14). Combining these two as binary (yes/no) characteristics, gives four groups that define the need for care and support, as inspired by the clientship-model (13, 15, 16) (Figure 1). Especially the group with complex health problems as well as limited resources forms a challenge for society and health and social care provision. This group presents a frail population, with a high care need and with a high risk of developing negative health-related events. Most studies on forecasts focus on the development of health but we think it is also important to explore the forecasts on the availability of resources. The aim of this study was to obtain insight into how the 65+ population is distributed across the four groups of need for care and support in 2020, and the expected distribution in 2040, and to identify the developments that may affect the future distribution.

## Data and methods

This study consisted of three parts. First, we estimated the distribution of the 65+ population for the year 2020 across the following groups; (1) complex health problems and sufficient resources, (2) complex health problems and limited resources, (3) no complex health problems and sufficient resources and (4) no complex health problems and limited resources. Second, we estimated this distribution for 2040 based on expected demographic developments only, assuming that the proportion in each group would remain the same. Third, we estimated this distribution for 2040 based on expert opinion using a two-stage Delphi study. With this study, we also assessed the expected developments that may drive the future changes in distribution, and the level of consensus on the direction these developments might take.

### Estimates for 2020

The distribution of the population across the four groups of need for care and support based on the prevalence of complex health problems and the availability of resources to manage these was estimated for the year 2020 using age-sex specific data from three population-based studies.

The Nivel’s Primary Care Database (Nivel Zorgregistraties eerste lijn) (17) uses routinely recorded data from healthcare providers to monitor health and health services utilization in a representative sample of the Dutch population. Diagnoses in primary healthcare are registered according to the International Classification of Primary Care (ICPC) (18). This data source provided data on age-sex specific prevalence of complex health problems for those aged 65+. The data compromises electronic medical records of patients from approximately 10% of general practices in the Netherlands (*n* = 1.331.882, of which 253.309 is 65+).

The National Health Monitor of the Netherlands (2016) (19) provided data on the age-sex-specific prevalence of having limited health resources for those aged 65+. It is a survey that aims to collect national, regional and local data on health, social situation and lifestyle. Municipal Health Services distribute this survey in collaboration with the National Institute for Public Health and the Environment (RIVM) commissioned by the Ministry of Health (*n* = almost 460,000 with a response rate of 40%).

The 6th measurement round of the Doetinchem Cohort Study (DCS) (20) provided data on: the age-sex specific prevalence of complex health problems and presence of resources in the age group 65–85 years (*n* = 3,500). The advantage of this dataset for these analyses is that both data on diseases, disability and resources are available (21).

The operational definition of ‘complex health problems’ was as close as possible to ‘those having at least conditions from two of the following clusters of disease: cardiovascular/metabolic disease, respiratory/musculoskeletal disorders, depression, visual or hearing problems, cancer or severe neurological disease. The operational definition of limited resources to social.

problems was based on at least two of the following characteristics: Living alone, low educational level, receiving informal care, inability to meet basic needs and insufficient self-reliance.

### Estimates 2040 based on demographic projections

The age-sex specified prevalence of (the combination of) complex health problems and the availability of resources as determined in 2020 were applied to the age-sex specified population projections of 2040 to get estimates for 2040. These projections are published yearly online by Statistics Netherlands. We used the projections published in 2020 (4).

### Population estimation 2040 based on expert opinion: Delphi study

A Delphi consensus procedure was conducted between October 2020 and January 2021. Experts were invited to share, in a structured manner, their thoughts on the characteristics of the 65+ population of 2040 in terms of complex health problems and resources to manage these, and to identify the developments affecting these characteristics.

The Delphi methodology aims to systematically collect opinions from a group of experts and achieve consensus (22, 23) for topics where evidence is lacking. The use of anonymity of participants, iteration and feedback allows the participants to openly give their opinion and change their opinion during the process (24).

### Expert panel

A multidisciplinary panel of 39 experts were recruited *via* email from the networks of the research team. Fields of expertise were older people and health and social care in the Netherlands. The panel included policy makers, researchers, insurers, (advocates of)older people or people with dementia and health organization advisors.

### Procedure

The Delphi-process consisted of two rounds, each running for 3 weeks using the MeetingSphere and Formdesk electronic platforms. Both Delphi rounds consisted of completing an online questionnaire and participating in a guided discussion on the online platform. The responses to the questions were fed back anonymously to the participating experts before the discussion session. Experts could revisit the platform at any time during the discussion sessions and provide their suggestions or comments. The Delphi-study was anonymous for both the experts as well as the research team.

#### Delphi round 1

The first round of the Delphi-study was aimed at identifying developments affecting the occurrence of complex health problems and the availability of resources in the Netherlands in 2040. The project was introduced to the experts by showing an animation with an introduction of the conceptual model and the population distributions in 2020 and 2040 (demographic projections only) across the four groups of the clientship-model (13, 15, 16).

The questionnaire was pilot tested among four colleagues at the National Institute for Public Health and the Environment to make sure the questions and the procedure were clear to the participants. The questionnaire included questions on:

A. Complex health problems

- How will the future percentage of older people aged 65+ compare to the current percentage of people aged 65 +? (using a 5-point Likert scale ranging from a large decrease to a large increase)
- Which developments do you expect to affect the future percentage of older people with complex health problems? A list of 13 potential categories (number of people with at least one chronic condition, treatment options, culture, diagnosis. Infectious diseases, lifestyle and behavior, life expectancy, mental well-being, environment, psychosocial well-being, locus of control, vitality and working conditions) was presented, and participants were invited to add to this list.

B. Resources

- How will the availability of resources amongst older people in the future compare to the availability of resources amongst the older people now? (using a 5-point Likert scale ranging from a large decrease to a large increase)
- Which developments do you expect to affect the availability of resources amongst older people in the future? A list of 12 potential categories (digital skills, health literacy, household composition, income, living conditions, independence, informal care, social network, local amenities, healthcare staff, healthcare technology) was presented, and participants were invited to add to this list.

C. Complex health problems and resources combined

- How will the future distribution of older people according to complex health problems and the availability of resources compare to the current situation? The four groups [(A) without complex health problems and with sufficient resources, (B) without complex health problems and with limited resources, (C) with complex health problems and with sufficient resources and (D) with complex health problems and with limited resources] were presented, and participants were asked to rate each group (using a 5-point Likert scale ranging from a large decrease to a large increase).

Free-text boxes were available for all questions for experts to explain their choices.

#### Delphi round 2

The second round of the Delphi-study was aimed at elaborating on how all the developments identified in round 1 would affect the prevalence of complex health problems and the availability of resources among older people aged 65+ in the future. In addition, the participants were asked to give their expectation on the distribution of proportions across the four groups. The participants could divide a 100% across the four groups.

### Data analysis of the Delphi-study

Consensus was defined as a certain percentage of agreement. In this study, three levels of consensus were used. “High consensus” was defined as ≥75% agreement on the expected strength of the effect of a development. “Intermediate consensus” was defined as ≥62.5% agreement on the expected strength of the effect of a development. “Low consensus” was defined as <62.5% agreement. The criteria for consensus were determined before the start of the study based on literature using similar study designs (25, 26).

## Results

### Expert panel

From the 39 invited experts, 26 experts responded to the first questionnaire (response rate 67%). Sixteen experts responded to the second questionnaire (response rate 62%). Reasons for not participating or dropout were mainly time constraints.

### Estimations for 2020 and 2040

**Figure 2:**
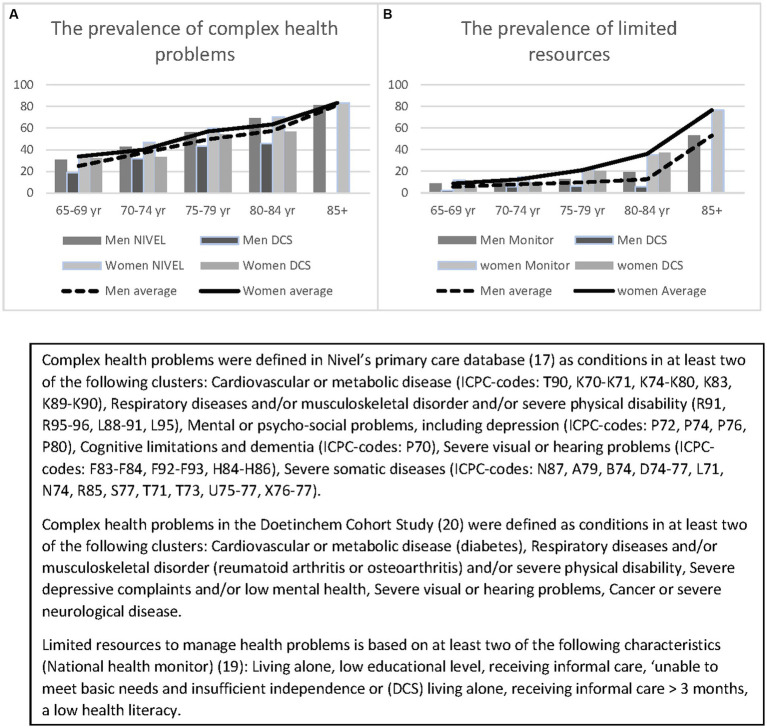
The prevalence of complex health problems **(A)** and limited resources **(B)** by sex and age: estimations and population.

The prevalence in 2020 of complex health problems was about 30% in people aged 65–69 years and about 80% for people aged 85 and above. The prevalence of older people with limited resources was 10 and 60%, respectively (Figure 2). All figures were higher among women compared to men.

**Figure 3:**
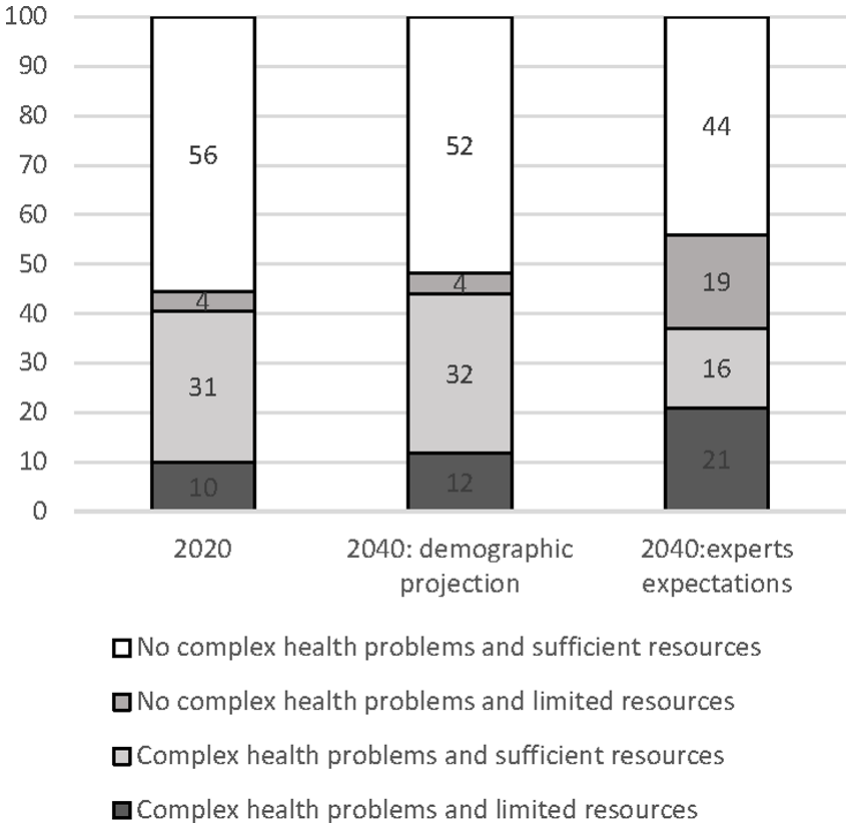
Distribution of the 65+ population across the four profiles in 2020 and 2040 based on demographic projections and experts’ expectations.

Using the age-sex-specific figures, the estimated proportion of the 65+ population with complex health problems and limited resources in the year 2020 was 10%, for complex health problems with sufficient resources 30.7%, for no complex health problems and limited resources 3.8% and for those without complex health problems or limited resources 55.6% (Figure 3). For 2040, the demographic forecasts show a shift to a larger population with complex health problems and limited resources of 11.7% mainly at the expense of those without challenges. The experts who took part in the Delphi study gave a different picture: taking all trends and developments into account the experts estimated the 65+ population with complex health problems and limited resources to be 22% (compared to 11.7%) and for those without complex health problems and limited resources 19% (compared to 4.1%).

### Developments affecting complex health problems and resources

In round 1 of the Delphi-study two developments were added in relation to complex health problems and one development in relation to resources. A total of fifteen developments potentially affecting the future prevalence of complex health problems were identified (Table 1) and thirteen developments affecting the availability of resources among the 65+ in 2040 (Table 2). Explanations provided by the experts on the assumed trend for each development are reported in Tables 1, 2.

**Table 1:** Trends affecting the prevalence of complex health problems among 65+ the period 2020–2040, as expected by experts found with a Delphi study.

In the next 20 years…			The total percentage of older people with complex health problem will…		
	Direction	Consensus	Direction	Consensus	Explanation
The number of people with at least one chronic condition	Increase	88%	Increase	81%	Ageing. Increase of life expectancy. Increase of obesity.
Life expectancy	Increase	81%	Increase	81%	Life expectancy increases faster than healthy life expectancy. Better healthcare techniques and more prevention.
Treatment options	Increase	100%	Increase	69%	More treatment options and technology.
Diagnostics/early detection of diseases	Increase	94%	Increase	69%	More early detection and better diagnostics ➔ increase in prevalence of chronic diseases ➔ increase in the number of people with complex health problems and decrease of mortality risk.
The influence of infectious diseases (relative to pre-corona)	Unchanged	69%	Unchanged	69%	The impact of infectious diseases (COVID-19) will remain and can lead to chronic diseases.
Mental health problems	Unchanged	63%	Unchanged	69%	
Self-management of health and care	Increase	81%	Unchanged	63%	
Socio-economic inequality	Increase	81%	Increase	63%	The gap between high and low SES is widening. Houses, school systems, digitalization play a role in this. Not everything is available to everyone in healthcare.
The influence of the environment on health (e.g. climate change)	Increase	69%	Increase	50%	More awareness and possibly increasing tension on social topics. At the same time there will be more technological advancements which creates solutions.
Cultural diversity	Increase	75%	Increase	44%	Links to the acceptance of new care options, techniques, healthy lifestyle and access to knowledge.
Vitality of older people	Increase	75%	Unchanged	44%	Increase will mainly be for a part of the population.
Manufacturability of life	Increase	56%	Unchanged	75%	
Working conditions (including workload, workload)	Deteriorate	44%	Unchanged	63%	The increased number of flexible jobs leads to more uncertainty and stress. This can lead to staff shortages and more work pressure. Digitalization might provide relief.
Psychosocial circumstances (e.g. stress, loneliness)	Unchanged	50%	Unchanged	56%	Increasing awareness of psychosocial circumstances.
Lifestyle and behaviour	Unchanged	44%	Unchanged	44%	In some aspects habits and behaviour will improve in regard to healthy lifestyle. In other aspects it will deteriorate. The gap between people with improvements and people with a deterioration will be larger.

Blue, high consensus among experts; green, intermediate consensus; orange, low consensus, including explanations provided by the experts on the assumed trend (2020, Netherlands).

**Table 2:** Trends affecting the presence of resources to manage complex health problems among 65+ in the period 2020–2040 as expected by experts found with a Delphi study.

In the next 20 years…			
	Direction	Consensus	Explanation
The number of one-person-households amongst older people	Increase	100%	More divorces which results in an increase of the number of one-person-households.
Availability of informal care providers	Decrease	100%	The demand for informal care will increase but there will be less supply of informal care.
Digital skills amongst older people	Increase	94%	The expectation is that older people will be (more) digitally skilled. But this should not be overestimated; digital developments move faster than attaining the skills. Moreover, skills should be maintained through time. In addition, the need for older people to be digitally skilled increases. As it is necessary for arranging their affairs, finding care and for their independence.
Technological possibilities	Increase	93%	More possibilities through health technology and e-health. However, its use depends on the availability, affordability and usability. There will be a gap between the possibilities and desirability.
Health and social care workforce	Decrease	93%	Ageing; higher demand for care and less people that can provide care. The professions are not attractive. Shortages in certain regions.
Health skills	Increase	79%	Increasingly, demands are made on self-reliance. Despite this increase, part of the older people will not be able to keep up.
Self-reliance	Increase	71%	People are increasingly called on being self-reliant. Self-reliance increases because of a higher education level. And with this the access to knowledge, health and healthcare increases. However, part of the older people will lag (low education level, language barriers, low socio-economic status).
Educational level	Increase	64%	The average education level increases. Which means that older people will on average have more resources available, such as a high income, health skills. But this does not apply for all older people, such as older people with a migrant background or people with low-or limited-income insurance like self-employed workers.
Variation in the supply of person-centred care	Increase	64%	Technology stimulates person-centred care.
Availability of suitable housing	Increase	57%	Increase in suitable houses, however it will not be sufficient for all older people.
Income and wealth	Unchanged	36%	On one hand the incomes will be higher, on the other hand the care will be more expensive. There will be a larger difference between the poor and rich.
Availability of a social network	Decrease	43%	On one hand the social network will decrease because people are having less children, living more often alone and are becoming older. On the other hand, the social network will be more diverse, and digitalization will play a role.
Availability of services (grocery shops, care facilities etc.) in the neighbourhood	Unchanged	50%	This will depend on the region. There will be a shortage in shrinking areas. Digitalization plays a role; online ordering and e-health.

Blue, high consensus among experts; green, intermediate consensus; orange, low consensus, including explanations provided by the experts on the assumed trend (2020, Netherlands).

### Complex health problems in 2040

Consensus on the direction of change of the developments in the next 20 years was reached for 73% of the developments (53% high and 20% intermediate). Consensus was slightly lower on how these developments affect complex health problems (Table 1). The level of consensus among the experts increased throughout the rounds. In round one, more than half of the experts (56%) expected the percentage of older people with complex health problems to increase, 28% expected stability and 16% a decrease. In round two, the majority (81%) expected the percentage of older people with complex health problems to increase. Developments contributing directly or indirectly to an increase of the occurrence of complex health problems were: increased prevalence of chronic conditions, increased life expectancy, more treatment options and early detection of diseases, and increased socio-economic inequality. Experts also mentioned developments contributing directly or indirectly to a potential decrease of complex health problems; increased self-management of health, improved lifestyle and health behavior and increased self-efficacy. Even though the experts agreed that there will be an increase in cultural diversity and an increase in the vitality of older people in the next 20 year they did differ of opinion on how these developments will affect the presence of complex health problems.

### Resources to deal with complex health problems in 2040

Consensus on the direction of change was reached for nine (69%) out of thirteen resources. The level of consensus among the experts increased throughout the rounds. In round one, more than a third of the experts (37%) expected the overall availability of resources to increase, 32% expected stability and 26% a decrease. In round two, 22% of the experts expected an increase of the availability of resources to manage complex health problems, half of the experts expected a decrease. Developments contributing to the expected decrease are an increase of one-person households and a decrease in the availability of both formal and informal care providers. The experts mentioned that the increase of health literacy, digital skills, educational level and independency will apply only to a part of older adults. The experts mentioned that those with a low educational level, language barriers and low socio-economic position will stay behind, causing the gap between the rich and poor to increase. No consensus was reached on the developments in availability of suitable housing, income and wealth, availability of a social network and services in the neighborhood.

## Discussion

This study aimed to get insight into the characteristics of the 65+ population of 2040, compared to the 65+ population in 2020, using modelling exercises and input from experts by means of a Delphi study. After taking a number of developments into account, the experts in the Delphi study expected the proportion of adults aged 65+ with complex health problems and limited resources in the Netherlands to increase from 10 to 22% in 2040. Also, this expected percentage is higher than the expected proportion of adults aged 65+ in 2040 based on demographic developments (12%). Although this figure of 22% is only an estimate, it presents reason for concern because it indicates that, when taking various developments into account, the need for care will be higher in the future.

This study shows that besides demographic developments also other developments affect the occurrence of complex health problems and the availability of resources, such as socioeconomic changes, developments in lifestyle and health, changes in living and working conditions, the increase of one-person households and decrease of formal and informal care providers. People will more often have to rely on informal care from family and friends, which in turn will have implications for the living and working conditions of the informal care providers such as work interference or change in work status and an increase of emotional stress (27–29). This will most likely negatively impact the availability of informal care.

The various developments are also expected to interact with or even amplify one another, resulting in a larger proportion of the population with complex health problems and with limited resources to deal with them. For example, improved technological possibilities are expected to facilitate diagnostics and early detection of diseases, which in turn might lead to an increase in the number of people with complex health problems. At the same time, a higher life expectancy, with chronic diseases being less lethal, results in more older people having multiple diseases, which also results in an increased number of people with complex health problems.

In this study, many experts explicitly mentioned that they expected an increased gap between different population groups regarding health status. For example, both the group of vital older people as well as the group of frail older people will become larger. So, the size of groups at both ends of the ‘health continuum’ are increasing. Resulting in highly different care needs amongst different groups of older people. Furthermore, the health differences between socio-economic groups and groups from diverse cultural backgrounds are expected to increase. These developments are an additional reason for concern, because socio-economic and cultural background are associated with health status.

Earlier future studies aiming to inform policymakers show similar developments as the ones identified in our study. Also, studies from other countries (Japan, United States, United Kingdom) indicate to expect increased pressure on the health and social care system (30–33). For example a study shows that if recent mortality trends continue, more people in England and Wales will need palliative care by 2040 (30). Another study showed that the age-standardized prevalence of disease will remain constant resulting in an expanding number of older people with care needs (31). Both for Japan and for United States micro-simulation modelling studies show that the need for care amongst 65 + -population will increase (32, 33).

### Implications

The expectation is that health and social care needs in the 65+ population will be larger in 2040 than in 2020, and this expectation is seen regardless of the method used, expert opinions or modelling exercises. The aging population will result in a tremendous challenge in dealing with the health problems and in determining how to appropriately deliver care for older people of the future (34), while taking (cultural) background and surroundings into account. Integrated care is seen as a potential way to coordinate and provide care tailored to peoples’ needs and preferences and reduce inequalities while improving patient outcomes. This can help the health system to cope with the increasing need for care. (13, 35, 36).

To deal with the increasing care needs different initiatives and measures are needed across different levels (37–39). For example on health system level support is needed for informal care provides. At the level of the living and environment, it concerns suitable housing, good cooperation and coordination amongst care and welfare professionals (integrated care) (40). At societal level, it is about image formation (the older people are not only weak and in need of help, a large part is able to continue to cope), a different way of training professionals so they are better equipped to support citizens with complex health problems (41). Initiatives may for instance focus on the reducing the increase in complex health problems, e.g., with more or better prevention strategies, and on expanding the possibilities to (self) manage the health problems of old age – e.g., programs to reduce loneliness, increase health literacy, various innovations based on technology, and promoting ‘positive health’ with its emphasis on ‘the ability to adapt and self-manage’ (42, 43). In addition, For all the different initiatives and measures the effects on reduction of the SES gap should also be taken into account.

This study illustrates the importance of not taking only ‘health problems’ into account but also the resources to deal with these, which is also emphasized in the research field of population segmentation based on health care needs (44–46). The clientship model (13, 15, 16), where our thinking model was based on, is now used in Finland to segment the populations and to aid the thinking of care and support needs and how to organize these (47). In particular it is used to strengthen the links and collaboration between primary care and social care and between primary care and hospital care. Because our study suggest that there will be a large growth in the size of the 65+ population with limited resources to deal with health problems, these finding urge for the care and support systems for these.

### Strengths and limitations

A strength of this study is the use of a Delphi study besides demographic projections. Studies aiming to make prediction usually make use of demographic projection, however this provides only part of the picture. The Delphi study allowed to incorporate expert opinions and different perspectives to determine future demands and needs. A limitation of this study is that different data sources have been used for the prevalence of complex health problems and the availability of resources. There is no single data source for the prevalence of complex health problems in combination with the availability of resources. Therefore, we combined information from a large registry of reliable and sufficiently detailed diagnostic data, with findings from a large-scale survey, monitoring data on a broad range of factors referring to ‘resources’ and cohort data with both diagnostic data and information on resources to get an estimation of the year 2020. The 2040-estimations are obtained by using demographic projections only are straightforward, keeping current prevalence rates of complex health problems and availability of resources constant. However, unexpected developments such as the COVID-19 pandemic may affect future demographics and thus change the circumstances/conditions of future care. Population health and the future proportion of older people with complex health problems and limited resources are subject to other uncertain developments. So we used the input of experts by means of a Delphi method, of which the strength is the use of a diverse expert panel. A sufficiently broad variety of experts, which is a prerequisite for a valid Delphi (26) method, gave insights into what they expect the population of older people to look like in 2040. However, because the Delphi was fully anonymous no information was collected on which of the invited experts participated during the different rounds. Therefore the heterogeneity of the panel could not be confirmed throughout the different rounds. Some important perspectives may have been missed if the group of participating experts is biased. Another limitation is that the experts can all be biased in the same direction. Also, some experts mentioned that trying to visualize how complex health problems and availability of resources will evolve together, was maybe too complex a thought experiment. This might also have led to drop-out of experts after the first round of Delphi. It is further important to realize that forecasts and future estimations do not represent facts. Our results give a picture of how experts see the future, and which an how developments will play an important role in the future. This information can help in finding appropriate interventions or solutions that will help to be better prepared for the future care needs.

## Conclusion

This research suggests that it is likely that a substantial part of the future 65+ population will suffer from complex health problems and will not have sufficient resources to manage these problems. This proportion is expected to be substantially higher than is expected based on demographic developments only, which is often done. A variety of developments contribute to this increase. Together with large workforce shortages in health and social care, these developments represent large challenges for health policy and asks for a fundamental redesign of the health and social care system. Further research is needed to understand how the different developments interact and how these can be incorporated in the foresight of population health.

## Data availability statement

The raw data supporting the conclusions of this article will be made available by the authors, without undue reservation.

## Ethics statement

Ethical review and approval was not required for the study on human participants in accordance with the local legislation and institutional requirements. The patients/participants provided their written informed consent to participate in this study.

## Author contributions

SB, SP, and MR led the funding acquisition for the study. FB, SP, HH, RH, MR, and SB contributed to the conception and design of the study. SP and RH performed statistical analysis of the registry data and patient-reported data. FB and SB contributed to the data collection and analysis of the Delphi study. FB and SP wrote the first draft of the manuscript. All authors contributed to the article and approved the submitted version.

## Funding

The present study was funded by Strategic Research Program of RIVM, the Dutch National Institute of Public Health and the Environment, which is an agency of the Netherlands Ministry of Health, Welfare and Sport (VWS).

## Conflict of interest

The authors declare that the research was conducted in the absence of any commercial or financial relationships that could be construed as a potential conflict of interest.

## Publisher’s note

All claims expressed in this article are solely those of the authors and do not necessarily represent those of their affiliated organizations, or those of the publisher, the editors and the reviewers. Any product that may be evaluated in this article, or claim that may be made by its manufacturer, is not guaranteed or endorsed by the publisher.
